# Lumbar spinal stenosis treatment with aperius perclid interspinous system

**DOI:** 10.1007/s00586-012-2222-2

**Published:** 2012-03-20

**Authors:** M. F. Surace, A. Fagetti, S. Fozzato, P. Cherubino

**Affiliations:** Department of Orthopaedic and Trauma Sciences “M. Boni”, Universitas Studiorum Insubriae, Viale Borri 57, 21100 Varese, Italy

**Keywords:** Lumbar spinal stenosis, Neurogenic intermittent claudication, Laminectomy, Interspinous device, MRI

## Abstract

**Purpose:**

The purpose of this study is to report clinical outcome and imaging changes of percutaneous Aperius stand-alone implant in patients with degenerative lumbar spinal stenosis and neurogenic intermittent claudication, which did not respond to conservative treatment.

**Method:**

Between January 2008 and July 2010, 37 patients (20 males and 17 females) with mean age of 64.3 years underwent surgery for the onset of claudicatio spinalis with Aperius PercLID interspinous device (Medtronic). In all patients, the diagnosis was: foraminal stenosis, in one case (2.7 %) it was associated to a degenerative anterior listhesis (I grade), in three cases (8.1 %) it was associated to an intraforaminal disc herniation. The mean follow-up was of 18 months (range 2–35 months). The patients were evaluated through the Oswestry disability index, Zurich Claudication Questionnaire (ZCQ), VAS scales. In all cases were obtained preoperative and in postoperative radiographs and magnetic resonance imaging.

**Results:**

The VAS score decreased significantly after surgery: the patients presented a mean VAS of seven preoperatively and two postoperatively (*p* < 0.001).

The ZCQ score significantly decreased postoperatively, with an average reduction of 21.89 % (*p* < 0.001).

The ODI score as well showed a significant reduction postoperatively of an average 26.09 % (*p* < 0.001).

**Conclusion:**

Despite of the brief follow up, the preliminary results are encouraging, showing a significantly decrease of the disability parameters, a marked improvement of the function with the vanishing of the claudicatio spinalis and the following increase of the free interval during the walk. Aperius PercLID system seems to offer an alternative to the traditional decompression surgery.

## Introduction

Neurogenic intermittent claudication (NIC) secondary to lumbar spinal stenosis (LSS) is a degenerative disease prevalent in the population older than 50 years of age [1], with about 8 % of adult population affected by this pathology [2].

The typical hyperextension of the affected spinal segment, caused by the gradual loss of disc height, leads to the annulus bulging, facets hypertrophy, spondylolisthesis, and calcification of the ligamentum flavum [3]. The NIC is the clinical manifestation of the root ischemia caused by the repetitive compression of the spinal canal and foramina.

Patients with stability of symptoms are treated with conservative therapy first, reporting a success rate variable from 15 to 50 %. In the past, the failure of the conservative therapy has generally occurred 4–6 years after the onset of degenerative lumbar spinal stenosis symptoms, leaving decompression surgery as the only alternative treatment.

The decompression surgery, with or without fusion, is reported to be more effective than conservative care in terms of pain relief and patient satisfaction [4].

The minimally invasive spine surgery has grown in recent years with the goal of a limited surgical approach, can reduce the symptoms, minimizing complications and anatomic changes.

Several studies have evaluated the effectiveness of the interspinous Aperius™ PercLID™ in patients with degenerative lumbar stenosis and NIC unresponsive to conservative treatment. This disease is the most frequent indication for spine surgery in patients over 65 [5].

The purpose of this device is the decompression of neurological structures in the early stages of the disease, providing a good alternative to more invasive decompressive surgery. Aperius™ PercLID™ offers the advantage of a totally percutaneous system: unilateral short skin incision and fast surgical procedure. Nardi [6] proposed surgical treatment with Aperius™ PercLID™ to patients after 6 months of unsuccessful conservative treatment.

Patients with a history of permanent motor deficits, previous surgery to the affected vertebral level, multiple surgeries to the spine, lumbar instability, severe scoliosis or severe symptomatic lumbar stenosis of more than three levels are not considered ideal candidates for implantation of the device Aperius™ PercLID™.

The most important biomechanical effects of Aperius™ PercLID™ are a reduction in compression of the dural sac, a limited range of motion in extension, minimal effects on flexion and the absence of effects to adjacent intervertebral levels.

Purpose of this study is to report clinical outcome and imaging changes of the percutaneous Aperius stand-alone implant surgical technique in patients with degenerative LSS and NIC which did not respond to previously administered conservative treatment.

## Materials and methods

Between January 2008 and December 2010, 35 patients, 19 males (52.80 %) and 17 females with mean age of 64 years (range 45–88 years) underwent surgery for the onset of NIC with Aperius PercLID interspinous device (Medtronic) at the orthopedic and traumatology department of Varese Hospital.

We considered that all patients complained of a progressive low-back associated to radicular pain exacerbated by prolonged standing or by activities in the upright posture and relieved by a flexed position of the lumbar spine, an associated diminished walking distance capability.

Neurologic examinations were performed in all patients at the admission in hospital.

Every patient experienced conservative treatment consisting of medications to control pain and physical therapy without any benefit.

In all patients, the diagnosis was foraminal stenosis, in one case (2.7 %) it was associated to a degenerative anterior listhesis (grade I) according to the Meyerding grading system [5], in three cases (8.1 %) it was associated to an intraforaminal disc herniation that was removed during the same surgery. In 22 (59.4 %) patients, the involved level was L4–L5, in 15 (40.6 %) patients the treated levels were both L3–L4 and L4–L5.

Aperius stand alone was used in 1 level in 18 patients (51.4 %), 2 levels in 17 patients (48.6 %), for a total of 52 devices implanted. Aperius was placed at L3–L4 in 18 cases (34.6 %) and L4–L5 in 34 cases (65.4 %). They were 8 mm size devices in 5 (9.6 %), 10 mm in 15 cases (28.9 %), and 12 mm in 11 cases (6.8 %).

The implantation of Aperius was performed as an isolated procedure in 27 cases (72.9 %) and associated to another procedure in 8 patients (21.6 %): 6 herniectomy and 2 discectomy.

The mean follow-up was 23 months (range 8–40 months).

### Surgical technique

When the implantation of Aperius was performed as a stand-alone procedure, the surgery was performed under local anesthesia using Mepivacaine 2 % and Chirocaine. The patients were placed in flexed prone position. After the radiographic identification of the surgical level, a small incision is made parallel to midline at approximately 4–6 cm from the spinous processes. Under fluoroscopy, a trocar is introduced and advanced towards the selected interspinous space; the percutaneous insertion of increasing size dilators (8–10–12–14 mm devices are available) allows choosing the most appropriate trocar size able to achieve the optimal decompression.

### Clinical assessment

Clinical outcome was assessed by means of Visual Analog Scale (VAS) score for the assessment of low-back pain and leg pain, Zurich Claudication Questionnaire (ZCQ) [7], and the Oswestry Low Back Pain Disability Questionnaire [8].

### Imaging assessment

Standard standing radiographs and magnetic resonance imaging were obtained pre- and postoperatively in all patients.

Magnetic resonance images were evaluated in the mid sagittal plane for anterior and posterior disk height, interspinous distance and also the disc degeneration according to Pfirrmann classification (Table 1) [9].

**Table 1:** Classification of lumbar intervertebral disc degeneration according to Pfirrmann’s classification

Grade	Classification of lumbar intervertebral disc degeneration
Grade I	The structure of the disc is homogeneous, with a bright hyperintense white signal intensity and a normal disc height
Grade II	The structure of the disc is inhomogeneous, with a hyperintense white signal. The distinction between nucleus and anulus is clear, and the disc height is normal, with or without horizontal gray bands
Grade III	The structure of the disc is inhomogeneous, with an intermediate gray signal intensity. The distinction between nucleus and anulus is unclear, and the disc height is normal or slightly decreased
Grade IV	The structure of the disc is inhomogeneous, with an hypointense dark gray signal intensity
Grade V	The structure of the disc is inhomogeneous, with a hypointense black signal intensity. The distinction between nucleus and anulus is lost, and the disc space is collapsed

MR images were also used to classify lumbar foraminal stenosis according to Lee grading system (Table 2) [10].

**Table 2:** MRI grading system for lumbar foraminal stenosis

Grade	MRI grading system for lumbar foraminal stenosis
Grade 0	Normal
Grade I	Mild degree of foraminal stenosis
Grade II	Moderate degree of foraminal stenosis
Grade III	Severe degree of foraminal stenosis

The measuring software Roman^®^ v.170 [Cook e Poullain (2002–2005, Institute of Orthopaedics, Oswestry, UK)] was used to quantify radiologic parameters. Radiographic measurements were carried out by two independent observers. The radiologic parameters were determined as follows:

- Anterior disc height (aDH) and posterior disc height (pDH) and interspinous distance (mm)
- Foraminal cross-sectional area (FA) (mm^2^)

The margins of the foramen were marked with the cursor, and the software Roman^®^ v.170 measured the cross-sectional area of the foramen.

### Statistics

Data were analyzed by means of SPSS 11.0 software (SPSS Inc., IL, USA).

Independent and paired samples *t* tests were employed for all parametric tested variables. Correlations were investigated by means of regression analysis. Significance was set at *p* < 0.05.

## Results

There was a significant improvement in the VAS scores for low-back and leg pain, and in ZCQ scores for symptom severity, physical function, patients’ satisfaction, and in quality of life express by the Oswestry Low Back Pain Disability Questionnaire.

The VAS score decreased significantly after surgery: the patients presented a mean VAS of 7 (range 2–9) preoperatively and 2 (range 0–7) postoperatively (*p* < 0.001).

The ZCQ score also significantly decreased postoperatively, with an average reduction of 21.89 % compared to preoperative values (*p* < 0.001).

The ODI score as well showed a significant reduction postoperatively of an average 26.09 % (*p* < 0.001) when compared to preoperative values.

### Anterior disc height

The average aDH measurement went from a preoperative value of 11.07 (range 4.82–18.30 mm) to 11.21 mm (range 4.30–17.92 mm) at last follow-up measurement. This difference was not statistically significant (Table 3).

**Table 3:** Radiologic measurement

	Anterior disc height (mm)	Posterior disc height (mm)	Interspinous distance (mm)	Foraminal cross-sectional area (mm^2^)
Preoperative values				
Mean	11.07	7.77	8.43	125.91
Maximum	18.30	12.90	12.55	177.30
Minimum	4.82	3.43	3.43	58.00
Postoperative values				
Mean	11.21	9.17	11.92	148.17
Maximum	17.92	14.94	15.43	244.00
Minimum	4.30	3.35	5.26	53.60

### Posterior disc height

The average pDH measurement significantly went from a preoperative value of 7.77 (range 3.43–12.90 mm) to 9.17 mm (range 3.35–14.96 mm) at last follow-up measurement. This difference was not statistically significant. The average height growth was 1.31 mm (17 %) (Table 3).

### Interspinous distance

**Fig. 1:**
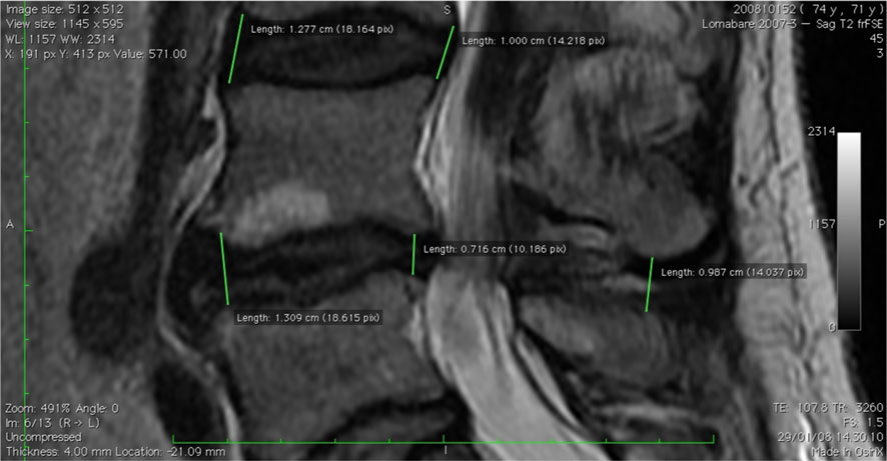
MRI preoperative measurement

**Fig. 2:**
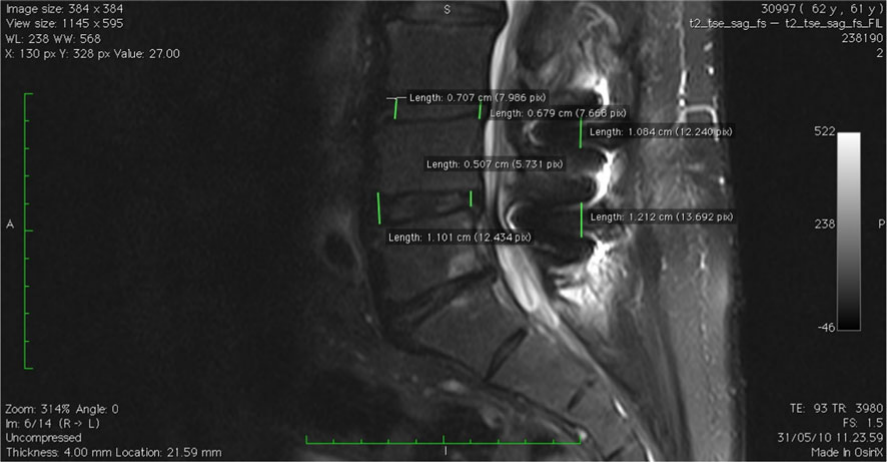
MRI postoperative measurement

The interspinous distance significantly increased from a preoperative 8.43 (range 3.43–12.55 mm) to 11.92 mm (range 5.26–15.43 mm) at follow-up. The average growth was 3.45 mm (41 %) (Table 3; Figs. 1, 2).

### Foraminal cross-sectional area (FA)

Foraminal cross-sectional area significantly increased from 125.91 (range 58.00–177.30 mm^2^) preoperatively to 148.17 mm^2^ (range 53.60–244.00 mm^2^) at last follow-up assessment (*p* < 0.001). The mean increase was 21.55 mm^2^, corresponding to 17.60 % of average FA (Table 3).

### Disc degeneration

As far as Pfirrmann classification is concerned, no variation in disc degeneration could be detected at follow-up evaluation in 65.40 % of the cases while 26.90 % worsened and only 7.70 % were improved. Statistical analysis showed that the difference in disc degeneration between the preoperative and the postoperative period was not significant (Table 3).

In one case (2.8 %) a treatment failure, defined as the need for a subsequent surgery to the level previously treated with Aperius™ PercLID™, occurred. The patient complained of a progressive worsening of pain symptoms over time. For this reason, at 4 months after surgery, new surgery was performed to remove the two interspinous devices implanted at L3–L4 and L4–L5 and a L3–S1 decompression and instrumented posterolateral fusion obtained. Despite clinical failure, at imaging interspinous devices were properly positioned.

Another case (2.8 %) showed a progressive worsening. Postoperatively, the patient had an improvement in clinical status, with a reduction of painful symptoms and restoration of normal daily activities. At a distance of 7 months postoperatively, the patient complained of recurrence of low back pain associated with radicular pain. For the progressive worsening of symptoms, the patient underwent surgery at another hospital.

One spinous process fracture occurred during implantation, in a severely osteoporotic patient.

**Fig. 3:**
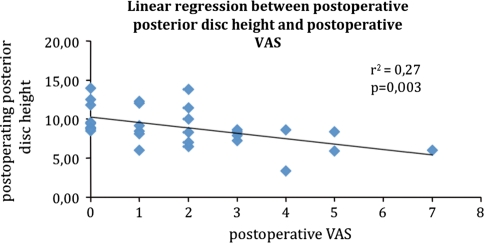
Linear regression between postoperative posterior disc height and postoperative VAS

A significant correlation (Fig. 3) was found between the average postoperative posterior disc height and the VAS, there was an inverse relation between these two parameters (*R* ^2^ = 0.27; *p* = 0.003).

A similar correlation was noted between the disc degeneration preoperatively and the satisfaction. The more degenerate the disc was the lower the satisfaction (*R* ^2^ = 0.30; *p* = 0.010).

## Discussion

As far as pain is concerned, statistical analysis showed a significant reduction after surgery in VAS mean score from seven preoperative to two postoperative points (*p* < 0.001), respectively. The interspinous device Aperius™ PercLID™ proves then, to be a valuable tool in achieving a reduction of painful symptoms complained by patients with degenerative lumbar stenosis, as demonstrated by the study of Nardi and Cabezas [6]. They reported a reduction of 37 % in postoperative VAS values for low back pain and pain radiating to the lower limbs. The present study achieved a far better pain reduction of 72 %.

As far as clinical outcome is concerned satisfactory, significant improvements were obtained for both ZCQ and ODI. Particularly, the ZCQ score significantly decreased postoperatively of an average 21.89 % points compared to preoperative values, confirming data reported by Nardi and the ODI, that was not considered in that study showed in the present study an as well significant reduction of an average 26.09 %, confirming that regardless the employed scoring system clinical outcome was invariably improved.

According to Wiseman et al. and Sobottke, Aperius interspinous system provides effective improvement of stenotic symptoms, independently from the preoperative degree of central canal and foraminal stenosis, achieving an appropriate distraction and decompression [11, 12].

MRI compared dimensional changes before and after device implantation, in anterior and posterior disc height as well as interspinous process distance. The average aDH measurement went from a preoperative value of 11.07–11.21 mm at last follow-up measurement. This difference was not statistically significant, and it is reasonable because the interspinous device, implanted posteriorly, mostly affects that area. Consistently, the pDH significantly increased from the preoperative measurements of 7.77–9.17 mm at follow-up. The average height gain registered was 1.31 mm (17 %) that strengthens the theory of a mainly posterior distractive effect of interspinous devices. A significant correlation was found between the average postoperative posterior disc height and VAS. Obviously, the interspinous distance also significantly increased from average preoperative 8.43–11.92 mm at follow-up, with a mean growth of 3.45 mm (41 %). The foraminal cross-sectional area, whose main diameter is strictly dependent on the posterior height and respective interspinous distance showed its significant increase of 17.60 % in surface area from 125.91 preoperatively to 148.17 mm^2^ at last follow-up assessment.

Similarly, disc degeneration could reasonably influence disc height and as far as Pfirrmann classification is concerned, no variation in disc degeneration could be detected at follow-up evaluation in 65.40 % of the cases while 26.90 % worsened and only 7.70 % were improved.

Statistical analysis showed that the difference in disc degeneration between the preoperative and the postoperative period was not significant. This veritable observation could possibly be explained by the fact that interspinous devices are not effective in reverting degenerative processes occurring at the disc site, maybe except for less severe and younger patients. Unfortunately, no significant correlation between age and disc recovery could be detected, but it could be because of the small sample size. Mainly, its function could be defined as a disease stabilizer, preventing further rapid disc degeneration. A similar correlation was noted between preoperative disc degeneration and satisfaction at follow up: the mostly degenerate the disc was the lower the satisfaction (*R* ^2^ = 0.30; *p* = 0.010). The last two findings seem to confirm that IPDs’ field of application should be limited to not so severe cases. In addition, the fracture of the spinous process reported was due to severe osteoporosis: prevention should be based on preoperative DEXA scans in order to avoid surgery with percLID in major bone resorption.

Finally, the surgical technique is easy and implies low morbidity, short hospitalization, and the possibility to obtain a neural decompression only through the extension limit given by the device, which is particularly helpful in the case of compromised patients. Despite the short follow-up results are promising and the success rate is comparable to decompressive laminectomy. Longer follow-up is mandatory to confirm these preliminary data and correctly assess the real efficacy of this device in the management of patients affected by degenerative lumbar stenosis.

## References

[1] Taylor VM, Deyo RA, Cherkin DC (1994). Low back pain hospitalization. Recent United States trends and regional variations. Spine.

[2] Hilibrand AS, Rand N (1999). Degenerative lumbar stenosis: diagnosis and management. J Am Acad Orthop Surg.

[3] Arbit E, Pannullo S (2001). Lumbar stenosis: a clinical review. Clin Orthop Relat Res.

[4] Atlas SJ, Keller RB, Wu YA (2005). Long-term outcomes of surgical and nonsurgical management of lumbar spinal stenosis: 8 to 10 year results from the Maine lumbar spine study. Spine.

[5] Metz LN, Deviren V (2007). Low-grade spondylolisthesis. Neurosurg Clin N Am.

[6] Nardi P, Cabezas D, Rea G (2010). Aperius PercLID stand alone interspinous system for the treatment of degenerative lumbar stenosis: experience on 152 cases. J Spinal Disord Tech.

[7] Stucki G, Daltroy L, Liang MH, Lipson SJ, Fossel AH, Katz JN (1996). Measurement properties of a self-administered outcome measure in lumbar spinal stenosis. Spine.

[8] Fairbank JC, Pynsent PB (2000). The Oswestry Disability Index. Spine.

[9] Pfirrmann CW, Metzdorf A, Zanetti M, Hodler J, Boos N (2001). Magnetic resonance classification of lumbar intervertebral disc degeneration. Spine.

[10] Lee S, Lee JW, Yeom JS, Kim KJ, Chung SK, Kang HS (2010). A practical MRI grading system for lumbar foraminal stenosis. AJR Am J Roentgenol.

[11] Sobottke R, Schlüter-Brust K, Kaulhausen T, Röllinghoff M, Joswig B (2009). Interspinous implants (X-Stop, Wallis, Diam) for the treatment of LSS: is there a correlation between radiological parameters and clinical outcome?. Eur Spine J.

[12] Wiseman CM, Lindsey DP, Fredrick AD, Yerby SA (2005). The effect of an interspinous process implant to facet loading during extension. Spine.

